# Uneven chances of breastfeeding in Spain

**DOI:** 10.1186/1746-4358-7-22

**Published:** 2012-12-27

**Authors:** Isabel Río, Álvaro Luque, Adela Castelló-Pastor, María del Val Sandín-Vázquez, Rosana Larraz, Carmen Barona, Mireia Jané, Francisco Bolúmar

**Affiliations:** 1 National Centre of Epidemiology, Institute of Health Carlos III, Madrid, Spain; 2 Faculty of Sciences, University of Alicante, Alicante, Spain; 3 Department of Public Health Sciences, Faculty of Medicine, University of Alcalá, Madrid, Spain; 4 Department of Health, General Directorate of Public Health, Generalitat Valenciana, Valencia, Spain; 5 Division of Environmental and Reproductive Epidemiology, Spanish Network for Research in Epidemiology and Public Health (CIBERESP), Valencia, Spain; 6 Department of Health, Mother and Child Health Program, General Directorate of Public Health, Barcelona, Catalonia, Spain

**Keywords:** Feeding behavior, Perinatal care, Quality of health care, Determinants of breastfeeding, Method of birth

## Abstract

**Background:**

No large scale studies on breastfeeding onset patterns have been carried out in Spain. This work aims to explore the prevalence and the risk factors for not initiating breastfeeding in hospitals from Catalonia (CAT) and Valencia (V), two regions accounting approximately for 30% of the annual births in Spain.

**Methods:**

The prevalence of not initiating breastfeeding was calculated by maternal/neonatal characteristics and type of hospital, and logistic regression models were used to estimate crude and adjusted risks of not breastfeeding in each region.

**Results:**

Prevalence of breastfeeding initiation was 81.7% and 82.5% in Catalonia and Valencia, respectively. We identified conspicuous regional differences in the adjusted-risk of not breastfeeding, especially for multiple [CAT = 3.12 (95% CI: 2.93, 3.31), V = 2.44 (95% CI: 2.23, 2.67)] and preterm and low birth weight deliveries [very preterm and very low birth weight: CAT = 7.61 (95% CI: 6.50, 8.92), V = 4.03 (95% CI: 3.13, 5.19); moderate preterm and moderate low birth weight: CAT = 4.28 (95% CI: 4.01, 4.57), V = 2.55 (95% CI:2.34, 2.79)].

**Conclusions:**

Our results suggest the existence of regional variations in breastfeeding initiation in Spain. Taking into account the known short and long-term benefits of breastfeeding it is recommended that further research should explore these differences in order to prevent potential inequities in neonatal, child and adult health.

## Background

There is strong evidence on the short and long-term benefits of breastfeeding for the health of newborns. International institutions prompt the need of monitoring newborn’s nutrition trends and to promote early initiation of breastfeeding and continuation of breastfeeding during the first six months of life. A nation-wide registry of breastfeeding patterns does not yet exist in Spain. In 2006, the latest *National Survey of Health* showed that the estimated prevalence of breastfeeding in children from 6 months to 4 years was 68.4% at 6 weeks after birth and 52.48% and 24.72% at 3 and 6 months, respectively [1]. No large scale studies on the patterns and determinants of breastfeeding onset in hospital have been carried out in the country. Here we investigate the prevalence and the risk factors for not initiating breastfeeding in two Spanish regions.

## Methods

**Figure 1:**
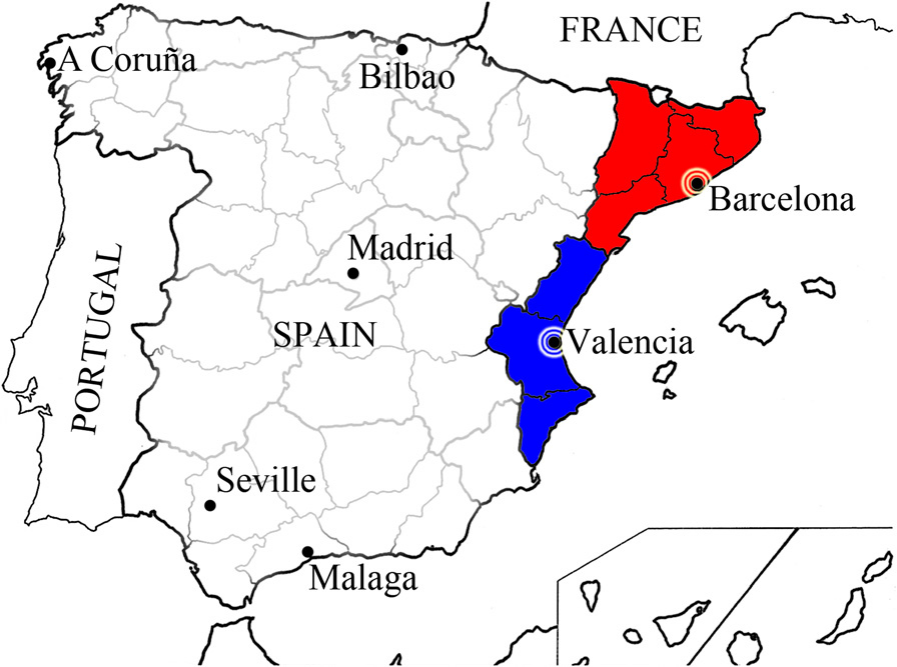
Map of Spain with Catalonia (red) and Valencia (blue) highlighted.

A cross-sectional study was carried out using data of births in 2005 and 2006 provided by the regional registries of metabolic disorders from Catalonia and Valencia (Figure 1), two Spanish regions accounting approximately for 30% of the total annual births in the country. These registries record information on childbirths (universal coverage) supplied by all public and private hospitals in each region. In addition to the information on metabolic disorders in the newborns they also record the type of nourishment initiated in the first 24 hours after delivery (breastfeeding vs. artificial feeding). Such information provides the only source allowing for monitoring of regional patterns of breastfeeding initiation in Spain. Data about type (single vs. multiple) and mode (vaginal vs. Caesarean section) of delivery and type of hospital (public vs. private), as well as information on maternal age and country of origin, and newborn’s characteristics such as sex, gestational age and birth weight are also registered.

We classified newborns into three categories according to the mother’s age (<20, 20-34 or ≥ 35 years) and in eight categories according to their region of origin (Spain, Latin-America, Maghreb, Eastern-Europe, Sub-Saharan Africa, North-Asia, South-Asia and “Other regions” which includes women belonging to countries in regions with minor representation in Spain). Regarding newborn’s characteristics, we grouped both gestational age and birth weight into three categories (very preterm (VPTB): < 32 weeks, moderate preterm (MPTB): 32-36 weeks, and at term: ≥37 weeks) and (very low birth weight (VLBW): < 1500 gr., moderate low birth weight (MLBW): 1500-2499, and normal weight: ≥ 2500 gr.). Percentages of not initiating breastfeeding by maternal and neonatal characteristics and by type of hospital were calculated for each one of the regions and compared using chi-square tests. Multiple logistic regression models were used to estimate crude and adjusted risk (odds ratios and 95% confidence intervals) of not initiating breastfeeding in each region. In order to estimate these risks for the most common adverse reproductive outcomes a variable combining birth weight and gestational age data was created and newborns classified into seven categories (VPTB but not VLBW, VLBW but not VPTB, VPTB and VLBW, MPTB with normal weight, MLBW with normal gestational age, MPTB and MLBW and normal gestational age and weight at birth). Analyses were carried out with the SPSS software version 17.0.

## Results

The number of registered births were 260,465, 62.4% of them in Catalonia and 37.6% in Valencia. Information on type of feeding was missing for 0.4% of births in Catalonia and 6.1% of births in Valencia. The total number of births and percentages by neonatal and maternal characteristics and type of hospital in each region are showed in Table 1. As expected, single and vaginal deliveries of 20-34 years old women in public hospitals and newborns at term and with normal weight were the most frequently reported. However, inter-regional comparisons showed a higher frequency of multiple deliveries and Caesarean sections in Catalonia than in Valencia, as well as a higher proportion of deliveries from immigrant women and deliveries in private hospitals. In contrast, percentages of prematurity and low birth weight were higher in Valencia than in Catalonia, as well as higher percentages of deliveries from older mothers. Overall, the lack of breastfeeding initiation was slightly higher in Catalonia (18.3% vs. 17.5%).

**Table 1:** Births in two Spanish regions by neonatal/maternal characteristics and type of hospital

	**Catalonia**	**Valencia**
	**n (%)**	**n (%)**
**Type of delivery**		
Single	144898 (95.9)	94688 (96.7)
Multiple	6269 (4.1)	3275 (3.3)
**Mode of delivery**		
Vaginal	111915 (71.5)	72593 (74.8)
Caesarean section	44588 (28.5)	24475 (25.2)
**Sex**		
Male	82705 (51.7)	45999 (51.3)
Female	77396 (48.3)	43679 (48.7)
**Gestational age (weeks)**		
VPTB (<32)	1329 (0.8)	697 (0.7)
MPTB (32-36)	10400 (6.6)	7325(7.8)
At Term (≥37)	146355 (92.6)	85430 (91.4)
**Birthweight (grams)**		
VLBW (<1500)	1237 (0.8)	646 (0.7)
MLBW (1500-2499)	10781 (6.6)	6931 (7.3)
Normal (≥ 2500)	150131 (92.6)	87755 (92.1)
**Maternal age (years)**		
<20	3269 (2.1)	1985 (2.4)
20-34	117436 (74.3)	59702 (71.0)
≥35	37325 (23.6)	22407 (26.6)
**Maternal origin**		
Spain	117760 (72.5)	74185 (75.7)
Latin-America	11869 (7.3)	5332 (5.4)
Maghreb	9921 (6.1)	2544 (2.8)
Eastern-Europe	3366 (2.1)	3588 (3.7)
Sub-Saharan Africa	1830 (1.1)	487 (0.5)
North-Asia	1718 (1.1)	441 (0.5)
South-Asia	1287 (0.8)	160 (0.2)
Other regions	2707 (1.7)	1776 (1.8)
**Type of hospital**		
Public	115557 (71.1)	86858 (89.5)
Private	46945 (28.9)	10141 (10.5)
**Breastfeeding**		
Yes	132699 (81.7)	80835 (82.5)
No	29803 (18.3)	17128 (17.5)

Tables 2 and 3 shown, respectively, percentages and crude and adjusted risks of not initiating breastfeeding by maternal and neonatal characteristics and by type of hospital. A remarkable variability between regions was observed regarding the risk of not breastfeeding after multiple delivery or Caesarean section and also for preterm and low weight births. Thus, while the adjusted risk of not breastfeeding for women with multiple pregnancies was 2.44 (95% CI: 2.23, 2.67) in Valencia, this risk increased to 3.12 (95% CI: 2.93, 3.31) in Catalonia. The comparison of different levels of prematurity or low birth weight showed a finely graded association with breastfeeding in both regions, the more adverse reproductive outcome the higher the risk of not initiating breastfeeding, but these risks were always higher in Catalonia. Thus, while in Valencia the risk of not breastfeeding for MPTB or MLBW babies was 51% higher than for those at term and with normal birth weight [OR = 1.51 (95% CI:1.40, 1.61)], it was doubled in Catalonia [OR = 2.00 (95% CI: 1.90, 2.11)]. Newborns with both conditions had a 2.55 (95% CI:2.34, 2.79) higher risk of not breastfeeding in Valencia. but a 4.28 (95% CI:4.01, 4.57) higher risk in Catalonia. A similar pattern was identified for VPTB and VLBW neonates, with those presenting only one of these outcomes having a 2.73 (95% CI: 2.16, 3.44) higher risk in Valencia but 4.33 (95% CI: 3.84, 4.89) in Catalonia, and neonates with both conditions having the highest risk in both regions but, once again, clearly higher in Catalonia [OR = 7.61 (95% CI: 6.50, 8.92)] than in Valencia [OR = 4.03 (95% CI: 3.13, 5.19)]. The only factor leading to a higher risk of not initiating breastfeeding in Valencia than in Catalonia was the occurrence of a Caesarean section intervention, which increased 35% [OR = 1.35 (95% CI: 1.31, 1.39)] the risk of not initiating breastfeeding in Catalonia while doubling the risk in Valencia [OR = 2.09 (95% CI: 2.02, 2.18)]. Regarding other characteristics such as age and geographical origin of the mothers, the adjusted risk was higher for younger and older women and also higher for native than for foreign-born women in both regions. The magnitude of this difference was higher in Catalonia than in Valencia although confidence intervals of the regional risk estimations often overlap.

**Table 2:** Intra- and inter-regional comparisons of the percentages of not breastfeeding onset by neonatal/maternal characteristics and type of hospital

	**Catalonia**		**Valencia**		**2 Regions comparison**
	**n (%)**	**p-value**^**a**^	**n (%)**	**p-value**^**a**^	**p-value**^**a**^
**Type of delivery**		< 0.0001		< 0.0001	
Single	24060 (16.6)		15435 (16.3)		0.050
Multiple	3594 (57.3)		1693 (51.7)		< 0.0001
**Mode of delivery**		< 0.0001		< 0.0001	
Vaginal	17940 (16.0)		9787 (13.5)		< 0.0001
Caesarean section	10957 (24.6)		7098 (29.0)		< 0.0001
**Sex**		0.428		0.081	
Male	15254 (18.4)		7706 (16.8)		< 0.0001
Female	14156 (18.3)		7128 (16.3)		< 0.0001
**Gestational age (weeks)**		< 0.0001		< 0.0001	
VPTB (<32)	762 (57.8)		347 (49.8)		< 0.0001
MPTB (32-36)	4511 (43.4)		2528 (34.5)		< 0.0001
At Term (≥37)	23698 (16.2)		13443 (15.7)		0.202
**Birthweight (grams)**		< 0.0001		< 0.0001	
VLBW (<1500)	797 (64.4)		331 (51.2)		< 0.0001
MLBW (1500-2499)	4961 (46.0)		2495 (36.0)		< 0.0001
Normal (≥ 2500)	23978 (16.0)		13778 (15.7)		0.590
**Maternal age (years)**		< 0.0001		< 0.0001	
<20	530 (16.2)		329 (16.6)		0.729
20-34	20650 (17.6)		10041 (16.8)		< 0.0001
≥35	7746 (20.8)		4564 (20.4)		0.264
**Maternal origin**		< 0.0001		< 0.0001	
Spain	23709 (20.1)		14109 (19.0)		< 0.0001
Latin-America	1806 (9.1)		316 (5.9)		< 0.0001
Maghreb	747 (7.5)		149 (5.9)		0.004
Eastern-Europe	346 (10.3)		321 (8.9)		0.610
Sub-Saharan Africa	149 (8.1)		63 (12.9)		0.002
North-Asia	899 (52.3)		217 (49.2)		0.262
South-Asia	174 (13.5)		21 (13.1)		1
Other regions	335 (12.4)		342 (13.3)		0.260
**Type of hospital**		< 0.0001		< 0.0001	
Public	20271 (17.5)		15052 (17.3)		0.124
Private	9532 (20.3)		1899 (18.7)		< 0.0001

a = p-value < 0.05.

**Table 3:** Risk of not initiating breastfeeding in two Spanish regions by neonatal/maternal characteristics and type of hospital

	**Catalonia**		**Valencia**	
	**ORc**^**a **^**(CI 95%)**	**ORa**^**b **^**(CI 95%)**	**ORc**^**a **^**(CI 95%)**	**ORa**^**b **^**(CI 95%)**
**Type of delivery**				
Single	1	1	1	1
Multiple	6.75 (6.41, 7.11)	3.12 (2.93. 3.31)	5.50 (5.12, 5.90)	2.44 (2.23, 2.67)
**Mode of delivery**				
Vaginal	1	1	1	1
Caesarean section	1.71 (1.66, 1.75)	1.35 (1.31, 1.39)	2.62 (2.53, 2.71)	2.09 (2.02, 2.18)
**Gestational age/birth weight**				
≥37 weeks and ≥ 2500 grs.	1	1	1	1
MPTB or MLBW	2.45 (2.34, 2,57)	2.00 (1.90, 2.11)	1.90 (1.79, 2.01)	1.51 (1.40, 1.61)
MPTB and MLBW	6.63 (6.27, 7.01)	4.28 (4.01, 4.57)	4.10 (3.83, 4.38)	2.55 (2.34, 2.79)
VPTB or VLBW	6.00 (5.39, 6.69)	4.33 (3.84, 4.89)	4.69 (3.90, 5.64)	2.73 (2.16, 3.44)
VPTB and VLBV	10.37 (8.99, 11.96)	7.61 (6.50, 8.92)	6.29 (5.19, 7.62)	4.03 (3.13, 5.19)
**Maternal age**				
<20	0.91 (0.83, 1.00)	1.19 (1.07, 1.32)	0.98 (0.87, 1.11)	1.33 (1.17, 1.50)
20-34	1	1	1	1
≥35	1.23 (1.19, 1.26)	1.10 (1.06, 1.14)	1.27 (1.22, 1.32)	1.10 (1.06, 1.15)
**Maternal origin**				
Spain	1	1	1	1
Latin-America	0.40 (0.38, 0.43)	0.41 (0.38, 0.44)	0.27 (0.24, 0.30)	0.26 (0.23, 0.29)
Maghreb	0.32 (0.30, 0.35)	0.34 (0.32, 0.37)	0.27 (0.22, 0.31)	0.28 (0.24, 0.34)
Eastern-Europe	0.45 (0.41, 0.51)	0.48 (0.42, 0.54)	0.42 (0.37, 0.47)	0.43 (0.38, 0.49)
Sub-Saharan Africa	0.35 (0.30, 0.42)	0.36 (0.30, 0.43)	0.63 (0.49, 0.83)	0.55 (0.41, 0.73)
North-Asia	4.35 (3.96, 4.79)	5.54 (5.00, 6.15)	4.13 (3.42, 4.98)	4.71 (3.85, 5.77)
South-Asia	0.62 (0.53, 0.73)	0.60 (0.50, 0.71)	0.64 (0.41, 1.02)	0.60 (0.36, 0.98)
Others	0.56 (0.50, 0.63)	0.90 (0.84, 0.96)	0.86 (0.81, 0.91)	1.11 (0.99, 1.24)
**Type of hospital**				
Public	1	1	1	1
Private	1.20 (1.17, 1.23)	1.04 (1.01, 1.07)	1.10 (1.04, 1.16)	0.99 (0.93, 1.05)

*ORc*^*a*^ = Crude Odds Ratio.

*ORa*^*b*^ = Odds Ratio Adjusted for all the other characteristics.

## Discussion

This is the first large scale population-based study on breastfeeding onset patterns in Spanish hospitals and one of the few providing specific estimates of breastfeeding in preterm and low birth weight babies in European countries [2]. Comparison of Catalonia and Valencia, two regions with a wide representation of the total births in the country, indicated a very marked variability in the magnitude of the risk of not initiating breastfeeding imposed for known postnatal nutrition determinants. This variability implies that mothers in Catalonia face greater obstacles in initiating breastfeeding than their counterparts in Valencia when they have multiple pregnancies or when their newborns are very or moderate preterm and/or they have a very or moderate low birth weight. In contrast, women delivering by Caesarean section found increased barriers to maternal breastfeeding in this last region. Inter-regional differences also confirm the better rates of breastfeeding initiation among immigrants compared to native women, with the exception of North-Asiatic mothers as described recently [3]. However, according to our analysis, this advantage seems slightly reduced among immigrant groups living in Catalonia.

The probability of initiating breastfeeding is a complex function of individual, social, cultural and clinical factors. Moreover, there is an obvious challenge to feeding most vulnerable infants. However, a marked variability in feeding practices of neonatal intensive care units has been reported indicating that, even under these circumstances, breastfeeding can successfully be established [4-6]. Poor guidelines on the importance of breastfeeding and lack of adequate professional training in breastfeeding promotion strategies as well as inadequate practices in maternities and neonatal intensive units have been proposed as barriers for optimal breastfeeding rates [7,8].

Some caveats must be considered when evaluating the results presented here. Firstly, there are no validation studies of breastfeeding data reported by the registries of metabolic disorders and this represents an important limitation of our study. Secondly, registries do not provide information on a large number of factors associated with initiating breastfeeding and we cannot rule out their potential influences. Moreover, information about the type of feeding initiated in hospital is presented as a dichotomous variable and we cannot discriminate if regional differences identified here are on exclusive breastfeeding and/or mixed feeding. Finally, data collected in the registries refers to the type of feeding initiated during the next 24 hours after birth, which does not necessarily imply that newborns, especially very preterm and with very low weight at birth, have not started breastfeeding later before they leave the hospital. However, this possibility could equally affect both regions and would not explain inter-regional differences found here. We cannot rule out the putative influence of missing values in our results. In Valencia no information was available on the type of feeding provided to 6.1% of babies, while in Catalonia the missing values for this variable were only 0.4%. As a strategy to evaluate the potential influence of missing values in our results, we repeated the statistical analysis under the strong assumption that all preterm and low weight births in Valencia with missed data about the type of nourishment (15.6% and 13.7% of total cases with missing values for type of feeding) started artificial feeding. Despite a reduction in regional differences, the results of this second analysis (data not shown) indicated that even in this improbable scenario, estimates of risk still remain more favorable for very and moderate premature or low birth weight infants born in Valencia than for those born in Catalonia. Regarding the potential influence of missing values for other variables such as gestational age or birth weight, we confirm that the proportions of breastfeeding were consistent with our results. Thus, in Catalonia 19.0% of newborns without information on gestational age and 18.8% with unknown birth weight were not breastfed, while these percentages were 14.7% and 14.5% in Valencia, respectively. Therefore, we presume the validity of our results.

## Conclusions

Our results suggest that further research on the regional differences on initiation of breastfeeding in Spain as well as on the differences in breastfeeding initiation in women having Caesarean or vaginal deliveries and VLBW babies is needed.

## Competing interests

The authors declare that they have no competing interests.

## Authors’ contributions

IR designed the study. CB and MJ were responsible for the acquisition of initial data. ACP and AL performed the statistical analysis. IR wrote the first manuscript. RL, MVSV and FB collaborated in the interpretation of results and all co-authors revised critically the successive drafts and approved the final version of the article.
